# Nutritional status and dietary diversity of school-age children living with HIV: a cross-sectional study in Phnom Penh, Cambodia

**DOI:** 10.1186/s12889-020-09238-8

**Published:** 2020-07-29

**Authors:** Junko Yasuoka, Siyan Yi, Sumiyo Okawa, Sovannary Tuot, Makoto Murayama, Chantheany Huot, Pheak Chhoun, Sokunthea Yem, Kazuki Yuzuriha, Tetsuya Mizutani, Kimiyo Kikuchi

**Affiliations:** 1 Research and Education Center for Prevention of Global Infectious Diseases of Animals, Tokyo University of Agriculture and Technology, 3-5-8 Saiwai-cho, Fuchu-shi, Tokyo, 183-8508 Japan; 2 Saw Swee Hock School of Public Health, National University of Singapore and National University Health System, Singapore, Singapore; 3 KHANA Center for Population Health Research, #33, St 71, Tonle Bassac, Chamkarmon, Phnom Penh, Cambodia; 4 Center for Global Health Research, Touro University California, Valejo, CA USA; 5 Cancer Control Center, Osaka International Cancer Institute, 3-1-69 Otemae, Chuo-ku, Osaka-shi, Osaka, 541-8567 Japan; 6 Kawasaki City Dentists Association, 2-10-10, Sunago, Kawasaki, Kanagawa Japan; 7 National Pediatric Hospital, Cambodia, #100, Street 110, Teuk Laak 1 Commune, Toulkok District, Phnom Penh, Cambodia; 8 Graduate School of Systems Life Sciences, Kyushu University, 744 Motooka, Nishi-ku, Fukuoka, Japan; 9 Graduate Education and Research Training Program in Decision Science for Sustainable Society, Kyushu University, Motooka 744, Nishi-ku, Fukuoka-shi, Fukuoka, 819-0395 Japan

**Keywords:** Cambodia, Children living with HIV, Dietary diversity, Nutrition, Stunting, Wasting

## Abstract

**Background:**

HIV/AIDS continues to be a major public health concern for children. Each day, worldwide, approximately 440 children became newly infected with HIV, and 270 children died from AIDS-related causes in 2018. Poor nutrition has been associated with accelerated disease progression, and sufficient dietary diversity is considered a key to improve children’s nutritional status. Therefore, this study aims to 1) examine nutritional status of school-age children living with HIV in Phnom Penh, Cambodia, and 2) identify factors associated with their nutritional status, especially taking their dietary diversity into consideration.

**Methods:**

This cross-sectional study was conducted in May 2018 within the catchment area of the National Pediatric Hospital, Cambodia. Data from 298 children and their caregivers were included in the analyses. Using semi-structured questionnaires, face-to-face interviews were conducted to collect data regarding sociodemographic characteristics, quality of life, and dietary diversity. To assess children’s nutritional status, body weight and height were measured. Viral load and duration of antiretroviral therapy (ART) were collected from clinical records. Multiple logistic regression analyses were performed to identify factors associated with stunting and wasting.

**Results:**

Of 298 children, nearly half (46.6%) were stunted, and 13.1% were wasted. The mean number of food groups consumed by the children in the past 24 h was 4.6 out of 7 groups. Factors associated with children’s stunting were age (adjusted odds ratio [AOR] 2.166, 95% confidence interval [CI]: 1.151, 4.077), household wealth (AOR 0.543, 95%CI: 0.299, 0.986), duration of receiving ART (AOR 0.510, 95%CI: 0.267, 0.974), and having disease symptoms during the past 1 year (AOR 1.871, 95%CI: 1.005, 3.480). The only factor associated with wasting was being male (AOR 5.304, 95%CI: 2.210, 12.728).

**Conclusions:**

Prevalence of stunting was more than double that of non-infected school-age children living in urban areas in Cambodia. This highlights the importance of conducting nutritional intervention programs, especially tailored for children living with HIV in the country. Although dietary diversity was not significantly associated with children’s nutritional status in this study, the findings will contribute to implementing future nutritional interventions more efficiently by indicating children who are most in need of such interventions in Cambodia.

## Background

While there have been great achievements in reducing human immunodeficiency virus (HIV) infections worldwide, children are still at risk of new infections. According to UNAIDS, the number of new HIV infections among children has been falling continuingly since 2000. Due to the global scale-up of antiretroviral therapy (ART) and the success in preventing mother-to-child transmission, 1.6 million new child HIV infections were averted between 2008 and 2017. However, in 2018, among the estimated 37.9 million people living with HIV worldwide, 1.7 million were children under 15 years old. Each day, worldwide, approximately 440 children became newly infected with HIV, and 270 children died from AIDS-related causes [1].

Poor nutrition has been associated with impaired immunity and accelerated disease progression among children living with HIV [2–4]. Infected children are at risk of growth failure [5, 6] as well as developmental and behavioral challenges [7]. Progressive stunting is considered the most common abnormality in children who were infected with HIV perinatally. Deficiencies of several micronutrients such as Vitamin A have been identified as an important factor negatively associated with growth [4, 8].

Recent studies conducted in Asia and Africa reported that, although ART improved the growth of children living with HIV, adjunct nutritional interventions such as nutritional supplementation and nutritional counselling are needed to promote better growth of these children [5, 9, 10]. In fact, the World Health Organization (WHO) recommends that children living with HIV increase energy intake and maintain a balanced macronutrient distribution for optimal growth and nutrition [11]. A recent systematic review revealed that vitamin A supplementation for HIV-infected infants and children led to a 45% reduction in the risk of all-cause mortality and reduced the likelihood of all-cause morbidity by 31% [8].

Along with micronutrient supplementation, sufficient dietary diversity is considered a key to improving overall nutritional and health status of children living with HIV [12–14]. A number of recent studies have explored risk factors for undernutrition, using dietary diversity score developed by the Food and Agriculture Organization [15–20]. Dietary diversity score, which consists of a simple count of food groups that an individual has consumed over the preceding 24 h, is considered a proxy for nutrient adequacy of the diet of individuals [21]. Several studies in African countries have revealed that low dietary diversity was associated with malnutrition, while consumption of an adequate variety of food can improve general nutritional status [15, 19, 20]. In order to plan an effective intervention program aimed at improving feeding practices and dietary diversity of children living with HIV, it is crucial to understand their current nutritional status and dietary intake.

Although the majority of children living with HIV reside in sub-Saharan Africa, Cambodia was home for 3300 infected children aged 0–14 in 2018 [1]. Cambodia has been making tremendous strides in HIV prevention and control [22], which has led to the fact that almost all people living with HIV who knew their HIV status were receiving treatment in 2018 [1]. Further, several studies have revealed a variety of health issues faced by HIV-infected adolescents and adults in Cambodia and made recommendations to effectively tackle these issues [23, 24]. However, few studies have been conducted in Cambodia and other Southeast Asian countries to evaluate nutritional status and dietary intake of children living with HIV, especially those of school age. Therefore, this study aimed to 1) examine the nutritional status of school-age children living with HIV in Phnom Penh, Cambodia, and 2) identify factors associated with their nutritional status, especially taking their dietary diversity into consideration.

## Methods

### Study design and sites

This cross-sectional study was conducted in May 2018 as a baseline survey of a randomized controlled trial, which has been conducted to examine the effectiveness of a daily oral care intervention on the health status of children living with HIV [25]. The study site was within the catchment area of the National Pediatric Hospital, which is the only health facility providing pediatric ART in Phnom Penh.

### Study population and recruitment

Out of the participants of the randomized controlled trial, only school-age children infected with HIV were included in this study. The multiple-step recruitment process of the randomized controlled trial has been described in a recent paper [25]. Briefly, children 3 to 15 years of age living with HIV were identified from patient lists of the National Pediatric Hospital. In total, 1113 children were considered eligible for the randomized controlled trial. Selection criteria were (1) aged 3–15 years, (2) possession of a patient ID at the National Pediatric Hospital, and (3) having received ART for at least 3 months prior to the study. Of the eligible children, 337 were selected, using a computerised algorithm by a data analyst, who was not a primary member of the research team. Selected children were recruited for the trial and participated in the baseline survey.

For this study, data from 298 children and their caregivers were included in the analysis after excluding 31 children below school age (under 6 years old) and eight children with missing data related to dietary diversity. There was no statistically significant difference in socio-demographic characteristics between the 306 children (including eight children with missing data) and 298 children, who were included in the analysis (e.g. age: *p*-value = 0.981, viral load: *p*-value = 0.959, ART length: *p*-value = 0.898). The sample size was larger than the required sample size of 271, which was calculated using Raosoft online sample size calculator (an estimated population of children living with HIV in Phnom Penh of 1650, 30% rate of stunting, 5% margin of error, and 99% confidence interval (CI)).

### Data collection

#### Questionnaire

Face-to-face interviews were conducted with the selected children and their caregivers, using semi-structured questionnaires. The questionnaire for children consisted of items regarding socio-demographic characteristics, HIV symptoms, and overall health and oral health quality of life. The questionnaire for caregivers included questions regarding socio-demographic characteristics and dietary diversity. Both questionnaires were developed based on existing tools used in previous studies and an existing guideline [21, 26, 27].

Dietary diversity, a characteristic of the quality of the diet, was assessed using a dietary diversity questionnaire, which was developed by the Food and Agriculture Organization. It is a proxy for nutrient adequacy of the diet of individuals and consists of a simple count of food groups that an individual has consumed over the preceding 24 h [21]. In this study, caregivers were asked if their child had consumed the food items described in Table 1.

**Table 1:** Food groups used to measure dietary diversity score

Food group	Food lists included in questionnaire
Group 1: Grains, roots, and tubers	Porridge, bread, rice, noodles or other foods made from grains
	White potatoes, white yams, manioc, cassava or any other foods made from roots
Group 2: Legumes and nuts	Any foods made from beans, peas, lentils, nuts or seeds
Group 3: Dairy products	Milk, such as tinned, powdered or fresh animal milk
	Yogurt or drinking yogurt
	Cheese or other dairy products
Group 4: Flesh foods	Liver, kidney, heart or other organ meats
	Any meat, such as beef, pork, lamb, goat, chicken or duck
	Fresh or dried fish, shellfish or seafood
	Grubs, snails or insects
Group 5: Eggs	Eggs
Group 6: Vitamin A fruits and vegetables	Pumpkin, carrots, squash or sweet potatoes that are yellow or orange inside
	Any dark green vegetables
	Ripe mangoes (fresh or dried, not green), ripe papayas (fresh or dried), musk melon
	Foods made with red palm oil, red palm nut or red palm nut pulp sauce
Group 7: Other fruits and veges	Any other fruits or vegetables
Others (not counted in the dietary diversity score)	Any oil, fats, or butter or foods made with any of these
	Any sugary foods, such as chocolates, sweets, candies, pastries, cakes or biscuits
	Condiments for flavour, such as chillies, spices, herbs or fish powder

To examine children’s well-being related diet, oral health-related quality of life was measured using the Child Perceptions Questionnaire [27, 28]. The questionnaire has been validated in Cambodia, and contains 16 items across four subscales: oral symptoms, functional limitations, emotional well-being, and social well-being. Responses for each item were rated on a five-point Likert scale: “never” = 0, “once or twice”=1, “sometimes” = 2, “often” = 3, “every day or almost every day”=4. The total score ranged from 0 to 64, and a higher score indicates worse oral health-related quality of life.

Overall health-related quality of life was measured using Pediatric Quality of Life Inventory 4.0 (PedsQL™ 4.0), which has been validated for children living with HIV [26]. The inventory contains 23 items across four subscales: physical, emotional, social, and school functioning. The five-point Likert scale for each item was: “never” = 0, “almost never” = 1, “sometimes” = 2, “often” = 3, “almost always” = 4. Items were reversed scored and linearly transformed to a 0–100 scale (100, 75, 50, 25, and 0). The sum of all the items over the number of items answered on all the scales was calculated, and the total score ranged 0–100. A higher total score indicated a better quality of life.

#### Health status

Body weight and height were measured by nursing staff of the National Pediatric Hospital, Cambodia, using electronic scales and a stadiometer to examine children’s nutritional status. Clinical records of children, including viral load, duration of ART use, and history of opportunistic infections, were collected by research assistants from the registered documents of the National Pediatric Hospital.

### Data management and statistical analysis

Open Data Kit 2.0 was used to directly record study participants’ responses to questionnaires (available at https://opendatakit.org/use/2_0_tools/). Body weight, height, and clinical data were recorded on paper-based forms, and entered electronically by data management assistants at the National Pediatric Hospital.

To assess the dietary diversity of children, a dietary diversity score (DDS) was calculated by summing the number of food groups consumed by each child over the previous 24-h recall period. In the Infant and Young Child Feeding guidelines published by the United Nations, dietary diversity scores of pre-school children are dichotomized: children who had at least four of the seven food groups were considered to have adequately diversified dietary intake [29]. In our study, we dichotomized the score using median as there is no recommended cut-off points for our study population (school-age children) [16–21]. The median of the children’s dietary diversity scores was calculated to determine the cutoff point between lower and higher dietary diversity. Based on the median value, children who consumed less than five food groups were classified into a lower dietary diversity group, and five or more into a higher dietary diversity group.

For anthropometric data analysis, z-scores of height for age and body mass index (BMI) for age were obtained using WHO Anthro Plus software (available at http://www.who.int.growthref/tools/en). Children whose height for age z-score was below − 2 standard deviations (SD) were considered as stunted. Children whose BMI for age z-score was below -2SD were considered as wasted.

Children were assigned to socio-economic status quartiles based on household assets and housing characteristics determined by principal component analysis [30].

Descriptive statistics were conducted to examine children’s socio-demographic characteristics and dietary diversity. Two multiple logistic regression analyses were performed to identify factors associated with stunting and wasting. In each analysis, the following independent variables were included: age, sex, household wealth, duration of receiving ART, viral load, history of disease symptoms (based on clinical records of the past year), quality of life, oral health quality of life, and dietary diversity. There was no multicollinearity among the independent variables. Educational status was excluded from the regression analyses because most of the children (95.6%) were enrolled in formal school education. All statistical analyses were conducted using STATA 14.0.

## Results

A total of 298 children aged 6–15 living with HIV in Phnom Penh were included in the analyses for this study. Sociodemographic characteristics of the study participants are described in Table 2. Out of 298 children, nearly half (139, 46.6%) were stunted, and 39 (13.1%) were wasted. Caregivers of most of the children (89.4%) reported that their child was infected with HIV through mother-to-child transmission, one was infected through a medical accident, and the others had no idea about the transmission route (data not shown). The length of time the children had been receiving ART ranged from 3 to 191 months. The majority of the children (79.5%) had a history of disease symptoms such as fever, cough or diarrhea within the past year.

**Table 2:** Sociodemographic characteristics and health status of the study population (Total *n* = 298)

Characteristics	Overall (n = 298)		Stunted (*n* = 139)		Not stunted (*n* = 159)		*p*-value	Wasted (*n* = 39)		Not wasted (*n* = 259)		*p*-value
	n	%	n	%	n	%		n	%	n	%	
Age												
6–10	101	33.9	41	29.5	60	37.7	0.317	11	28.2	90	34.7	0.684
11–12	90	30.2	44	31.7	46	28.9		12	30.8	78	30.1	
13–15	107	35.9	54	38.8	53	33.3		16	41.0	91	35.1	
Sex (Male)	152	51.0	72	51.8	80	50.3	0.798	32	82.1	120	46.3	< 0.001
Education (None/drop out)	13	4.4	4	2.9	9	5.7	0.271	0	0.0	13	5.0	–
Wealth tirtiles												
Low	98	32.9	53	38.1	45	28.3	0.179	15	38.5	83	32.0	0.491
Middle	99	33.2	41	29.5	58	36.5		14	35.9	85	32.8	
High	101	33.9	45	32.4	56	35.2		10	25.6	91	35.1	
Viral load (detected)	90	30.2	46	33.1	44	27.7	0.309	13	33.3	77	29.7	0.648
Length of ART tirtiles												
3–62 months	102	34.2	53	38.1	49	30.8	0.255	12	30.8	90	34.7	0.856
63–104	97	32.6	46	33.1	51	32.1		14	35.9	83	32.0	
105–191	99	33.2	40	28.8	59	37.1		13	33.3	86	33.2	
History of disease (clinical record past one year)	237	79.5	117	84.2	22	13.8	0.063	30	76.9	207	79.9	0.665
QoL tirtiles												
Low	103	34.6	45	45.5	58	36.5	0.613	12	30.8	91	35.1	0.326
Middle	96	32.2	44	44.4	52	32.7		10	25.6	86	33.2	
High	99	33.2	50	50.5	49	30.8		17	43.6	82	31.7	
Oral Health QoL tirtiles												
Low	105	35.2	51	51.5	54	34.0	0.434	13	33.3	92	35.5	0.199
Middle	88	29.5	36	36.4	52	32.7		16	41.0	72	27.8	
High	105	35.2	52	52.5	53	33.3		10	25.6	95	36.7	
Dietary diversity												
0–4 groups	133	44.6	60	43.2	73	45.9	0.226	21	53.8	112	43.2	1.540
5–7 groups	165	55.4	79	56.8	86	54.1		18	46.2	147	56.8	

Children’s dietary diversity is shown in Table 3. During the past 24 h, the majority of the children consumed foods from Group 1 (98.3%), 4 (98.0%), 6 (81.2%) and 7 (72.8%). About half took Group 5 (56.7%), and a third took Group 3 (36.9%). Only 16.1% of the children had foods from Group 2. The mean number of food groups consumed by the children were 4.6 out of 7 groups (median = 5, interquartile range = 1, data not shown). Other foods that were not included in the dietary diversity score, including fats, sugary foods, and condiments for flavor, were taken by the majority of children (83.2%). More than half of the children (53.7%) had sugary food such as chocolates, cookies, and cakes. None of the food groups was significantly associated with children’s stunting and wasting status.

**Table 3:** Dietary diversity of the study population

Dietary diversity	Overall (n = 298)		Stunted (n = 139)		Not stunted (n = 159)		*p*-value	Wasted (n = 39)		Not wasted (n = 259)		*p*-value
	n	%	n	%	n	%		n	%	n	%	
Group 1: Grains, roots, and tubers	293	98.3	138	99.3	155	97.5	0.228	38	97.4	255	98.5	0.644
Group 2: Legumes and nuts	48	16.1	18	12.9	30	18.9	0.166	3	7.7	45	17.4	0.125
Group 3: Dairy products	110	36.9	48	34.5	62	39.0	0.426	15	38.5	95	36.7	0.830
Group 4: Flesh foods	292	98.0	135	97.1	157	98.7	0.321	38	97.4	254	98.1	0.793
Group 5: Eggs	169	56.7	77	55.4	92	57.9	0.668	23	59.0	146	56.4	0.760
Group 6: VA rich fruits and veges	242	81.2	116	83.5	126	79.2	0.354	32	82.1	210	81.1	0.885
Group 7: Other fruits and veges	217	72.8	104	74.8	113	71.1	0.468	27	69.2	190	73.4	0.589
Others	248	83.2	115	82.7	133	83.6	0.833	31	79.5	217	83.8	0.503

**Fig. 1:**
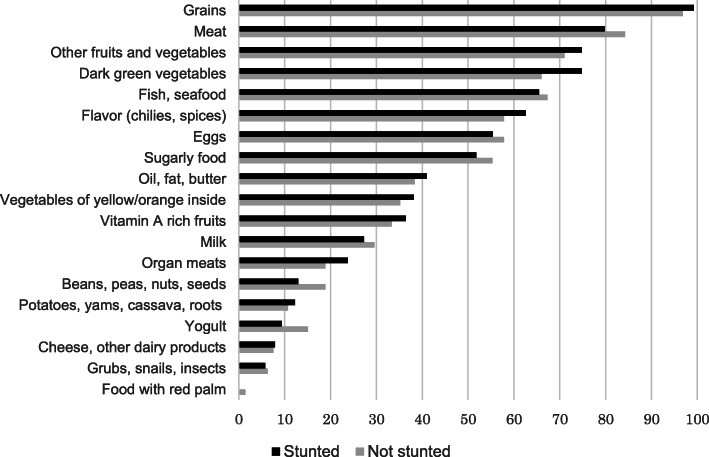
Food items that were consumed by the study population (%)

Detailed food items that were consumed by children are shown in Fig. 1. No significant differences were found in the consumption of each food item between stunted and not stunted children (data not shown).

Factors associated with children’s stunting were identified by multiple logistic regression analysis (Table 4). Children in the oldest age group (13–15) were 2.2 times more likely to be stunted compared to those in the youngest age group (6–10) (AOR 2.166, 95%CI: 1.151, 4.077). Children who belonged to the middle household wealth group were 46% less likely to be stunted compared to those in the lowest wealth group (AOR 0.543, 95%CI: 0.299, 0.986). Longer duration of receiving ART was significantly associated with less stunting: children who had received ART for the longest period (105–191 months) were 49% less likely to be stunted compared to those with the shortest ART duration of 3–62 months (AOR 0.510, 95%CI: 0.267, 0.974). Children who had disease symptoms during the past year were almost twice more likely to be stunted compared to those without any disease symptoms (AOR 1.871, 95%CI: 1.005, 3.480).

**Table 4:** Factors associated with stunting and wasting among the study population

Characteristics	Factors associated with stunting				Factors associated with wasting			
	AOR	95%CI			AOR	95%CI		
Age tertiles (Ref = 6–10)								
11–12	1.692	0.905	3.162		1.321	0.496	3.518	
13–15	**2.166**	1.151	4.077	*	1.849	0.711	4.807	
Sex (Male)	1.128	0.700	1.812		**5.304**	2.210	12.728	**
Wealth tirtiles (Ref = Low)								
Middle	**0.543**	0.299	0.986	*	0.946	0.396	2.256	
High	0.658	0.365	1.188		0.636	0.253	1.596	
Viral load (Detected)	1.195	0.699	2.044		1.066	0.481	2.363	
Length of ART tirtiles (Ref = 3–62 months)								
63–104	0.706	0.384	1.296		1.202	0.477	3.028	
105–191	**0.510**	0.267	0.974	*	1.074	0.405	2.843	
History of disease	**1.871**	1.005	3.480	*	0.983	0.406	2.379	
QoL tirtiles (Ref = Low)								
Middle	1.275	0.692	2.349		0.671	0.252	1.782	
High	1.447	0.752	2.785		1.441	0.566	3.669	
Oral Health QoL tirtiles (Ref = Low)								
Middle	0.657	0.346	1.251		1.702	0.672	4.312	
High	1.121	0.586	2.144		0.959	0.342	2.688	
Dietary diversity (Ref = 0–4 groups)								
5–7 groups	1.094	0.670	1.787		0.599	0.284	1.261	

*p < 0.05 **p < 0.01

Factors associated with wasting were also examined through multiple logistic regression analysis (Table 4). Only one factor was significantly associated with children’s wasting: male children were 5.3 times more likely to be wasted compared to female children (AOR 5.304, 95%CI: 2.210, 12.728).

## Discussion

This study examined nutritional status and dietary diversity of school-age children living with HIV in Phnom Penh, Cambodia. Nearly half of the children who participated in this study were stunted, and one-seventh were wasted. Factors associated with stunting were higher age, lower household wealth, shorter duration of receiving ART, and having disease symptoms during the past year. Only one factor, being male, was significantly associated with wasting. Dietary diversity, which was measured by a dietary diversity score, as well as any of the individual food items included in the score, were not associated with children’s nutritional status.

Prevalence of stunting was more than double among our study participants compared to non-infected school-age children living in urban areas in Cambodia. A recent study revealed that the overall prevalence of stunting among Cambodian children aged 6–17 years was 33.2% in 2014/2015, and that stunting was more prevalent in children living in rural areas than in children living in urban areas (36.4% vs. 20.4%, respectively) [31]. The high prevalence of stunting among our study participants coincides with a report indicating that HIV-infection was associated with growth delays in both weight and height [32, 33]. As children living with HIV are more seriously affected by malnutrition compared to non-infected children [2–4], implementing tailored nutrition programs, targeting children living with HIV, is highly recommended.

This study revealed that older age was associated with stunting, but that receiving ART for a longer duration could be a protective factor against stunting. This finding is similar to results of a recent study conducted in Thailand, which demonstrated that the younger the children at ART initiation, the greater the effect on height-growth velocity regardless of the ART regimen [34]. Another recent study revealed that HIV infection was associated with not only stunting but also low lumbar spine bone density among children living with HIV in Zimbabwe and that younger age at initiation of ART predicted high bone mineral density [35]. There are additional studies which stress the importance of initiating ART before irreversible stunting has occurred [36]. As the WHO and previous studies recommend, starting ART as soon as children are diagnosed with HIV and continuation of the treatment is crucial not only for the prevention of disease progression but also for prevention of growth retardation.

In this study, the dietary diversity score and any of the food groups and food items that constitute dietary diversity score, showed no significant associations with children’s nutritional status. On average, children consumed five out of seven food groups within the 24 h before the survey. This is slightly higher than four groups, which was reported by one of the few dietary diversity studies in Cambodia, targeting children living in rural areas in 2012 [37]. However, considering the high prevalence of stunting, improvement in both the quality and quantity of the diet of children living with HIV as well as relevant factors that support healthy dietary habit are vital. To identify growth delay as early as possible, it is recommended that nutritional assessment, including appetite and opportunistic infections, needs to be conducted [38]. Also, the WHO recommends that nutritional assessment and support should be an integral part of the care plan for children living with HIV [11]. Such support needs to include interventions to improve oral health as untreated dental caries and a delayed eruption of permanent teeth are associated with underweight and stunting among children in Cambodia, Indonesia and Lao PDR [39]. The timing of such nutritional interventions is also important: a recent study suggested that the first year on ART could be the best period to conduct nutritional interventions for children living with HIV to optimize their growth in the long term [40].

Findings from this study should be considered in the context of some limitations. First, because interview data were self-reported by children and their caregivers, there was a possibility of recall bias and courtesy bias. To minimize these biases, data were confirmed with clinical record whenever possible, and interviews were conducted by experienced and trained interviewers with on-site supervision. Second, dietary diversity was measured by questionnaire and no detailed exams such as micronutrient blood testing were conducted. Third, although it is common to dichotomize dietary diversity score of study population to evaluate individuals’ dietary diversity, small statistical difference between lower and higher dietary diversity groups could reduce the statistical likelihood of this variable being related to outcome variables, i.e. stunting and wasting. Therefore, to double check our results, we run other regression analyses, treating the dietary diversity score as a continuous variable. As a result, it was confirmed that dietary diversity of our study population was not significantly associated with stunting and wasting.

Despite these limitations, this is one of the few studies that examined nutritional status and dietary diversity of children living with HIV in Cambodia. Although dietary diversity was not associated with nutritional status, findings of this study will assist in strengthening future intervention programs, targeting children living with HIV in Cambodia and other resource-limited countries.

## Conclusions

This is one of the few studies examining nutritional status and dietary diversity of school-age children living with HIV in Cambodia. Prevalence of stunting among our study participants was more than double that of non-infected school-age children living in urban areas in Cambodia.

This highlights the importance of conducting nutritional intervention programs especially tailored for children living with HIV in the country. Although dietary diversity was not significantly associated with children’s nutritional status in this study, other factors such as age, household wealth, duration of receiving ART, and history of having disease symptoms during the past year were found to be important factors for stunting. These findings will contribute to implementing future nutritional interventions more efficiently by identifying children who are most in need of such interventions in Cambodia and other resource-limited countries.

## Data Availability

The datasets used and/or analysed during the current study are available from the corresponding author on reasonable request.

## Abbreviations

AOR: Adjusted odds ratio
ART: Antiretroviral therapy
CI: 95% confidence interval
DDS: Dietary diversity score
PedsQLTM 4.0: Pediatric Quality of Life Inventory 4.0
WHO: World Health Organization
